# Critique of: “Physical Activity Assessment Between Consumer- and Research-Grade Accelerometers: A Comparative Study in Free-Living Conditions”

**DOI:** 10.2196/mhealth.6860

**Published:** 2017-02-27

**Authors:** Jairo H Migueles, Cristina Cadenas-Sanchez, Francisco B Ortega

**Affiliations:** ^1^ Faculty of Sport Sciences PROFITH “PROmoting FITness and Health through physical activity” research group. Department of Physical Education and Sports University of Granada Granada Spain; ^2^ Karolinska Institutet Department of Biosciences and Nutrition Karolinska Institutet Huddinge Sweden

**Keywords:** Fitbit, activity tracker, actigraphy, physical activity, aerobic exercise, validity

In a recent issue in this Journal, Dominick et al., compared the outcome of a consumer-grade accelerometer against a research-grade accelerometer [1]. More specifically, they compared the Fitbit Flex (Charge and Surge) placed on the wrist against the GT3X (ActiGraph, Pensacola, USA, FL) placed on the hip. The authors observed large differences between methods, i.e. “Fitbit significantly overestimated METs for average daily activity, for overall minutes of reported exercise bouts, and for walking and run or sports exercises (all *P*-values <.001); and for average daily activity, Fitbit significantly underestimated the proportion of time in sedentary and light intensity by 20% and 34%, respectively, and overestimated time by 3% in both moderate and vigorous intensity (all *P*-values <.001)”.

We find a major problem in the design of the present study, with potential to largely affects its results and interpretation. The authors aimed to compare activity measured by two different devices. However, these two devices were attached to two completely different locations, i.e. wrist (Fitbit) vs. hip (GT3X). As a consequence, the differences observed in this study could actually be due to the different locations rather than the real differences between devices. It is well known that the same accelerometer when attached to the wrist register markedly more accelerations than when attached to the hip [2–4]. As expected, the authors observed a higher level of activity in the wrist-accelerometer than in the hip-accelerometer. If the authors wanted to compare a consumer-accelerometer with a research-accelerometer, which is a very interesting research question, they should have placed both devices (Fitbit and GT3X) on the same wrist. Large-scale studies such as the National Health Examination Survey, NHANES, are placing the GT3X accelerometer on the wrist. There are now available cut-points to classify accelerations from GT3X attached to the wrist into time spent in different intensities of physical activity [2,3], so it would have been fully correct methodologically to attach both devices to the wrist. The authors acknowledge as a limitation that accelerometers were placed in different locations. However, there is no explanation as to why they did so. Unfortunately, we will only be able to know how comparable these two accelerometers are when a future study places both of them on the same location.
